# Comparison of Factors Associated with Disordered Eating between Male and Female Malaysian University Students

**DOI:** 10.3390/nu12020318

**Published:** 2020-01-25

**Authors:** Yit Siew Chin, Mahenderan Appukutty, Masaharu Kagawa, Wan Ying Gan, Jyh Eiin Wong, Bee Koon Poh, Zalilah Mohd Shariff, Mohd Nasir Mohd Taib

**Affiliations:** 1 Department of Nutrition and Dietetics, Faculty of Medicine and Health Sciences, Universiti Putra Malaysia, UPM Serdang, Selangor 43400, Malaysia; chinys@upm.edu.my (Y.S.C.); wanying@upm.edu.my (W.Y.G.); zalilahms@upm.edu.my (Z.M.S.); nasir.jpsk@gmail.com (M.N.M.T.); 2 Research Centre of Excellence for Nutrition and Non-Communicable Chronic Diseases, Faculty of Medicine and Health Sciences, Universiti Putra Malaysia, UPM Serdang, Selangor 43400, Malaysia; 3 Faculty of Sports Science and Recreation, Universiti Teknologi MARA, Shah Alam, Selangor 40450, Malaysia; 4 Jeffrey Cheah School of Medicine and Health Sciences, Monash University Malaysia, Sunway, Selangor 47500, Malaysia; 5 Institute of Nutrition Sciences, Kagawa Nutrition University, Saitama 350-0288, Japan; mskagawa@eiyo.ac.jp; 6 Nutritional Sciences Programme & Centre for Community Health Studies, Faculty of Health Sciences, Universiti Kebangsaan Malaysia, Jalan Raja Muda Abdul Aziz, Kuala Lumpur 50300, Malaysia; wjeiin@ukm.edu.my (J.E.W.); pbkoon@ukm.edu.my (B.K.P.)

**Keywords:** disordered eating, Malaysia, university students, body image, depressive symptoms

## Abstract

Disordered eating is prevalent among university students, especially females. Whilst literature suggests that factors associated with disordered eating may differ according to gender, such an association has not been studied in Malaysia. This cross-sectional study aims to compare factors associated with disordered eating between male and female university students. A total of 716 university students (male: 27.4%; female: 72.6%) were recruited in Kuala Lumpur and Selangor, Malaysia. All participants completed a set of self-administered questionnaires and their body weight and height were recorded. About one in five of the university students (20.3%) were found to have disordered eating. There were more female students (22.9%) disordered eating compared to males (13.3%, χ^2^ = 8.16, *p* < 0.05). In male students (β = 0.228, *p* < 0.01), depressive symptoms were the only significant predictor for disordered eating. In females, the strongest predictor was depressive symptoms (β = 0.214, *p* < 0.001), followed by body size satisfaction (β = −0.145, *p* < 0.01) and body appreciation (β = −0.101, *p* < 0.05). These findings suggest that there are gender differences in the factors associated with disordered eating among Malaysian university students. Intervention programmes that address disordered eating should take into account these sex differences and its contributing factors.

## 1. Introduction

University students, specifically undergraduate students, are typically aged between 18 and 25 years and are therefore in a period of emerging adulthood [1]. Emerging adulthood has been identified as an important time for establishing and intervening on long-term health behavioural patterns [2,3]. During this period of emerging adulthood, they acquire and establish psychosocial attributes such as self-efficacy, which is associated with the beneficial health behaviours of young adults [4]. Self-efficacy is important at this life stage to provide support in long-term behavioural patterning. The national trend data from the United States suggest that emerging adulthood is a risky period for the development of obesity, as well as unhealthy diet and physical activity practices [5]. However, this age group is often being overlooked and understudied [5]. Substantial life-changing transitions occur when young adults complete secondary school and start university life, where many of them would have their first experience of living away from home [6,7,8]. Hence, university life is a critical period in their emerging adulthood to shape their eating attitudes and behaviour that may be associated with weight gain and poor health [9,10,11].

Body image is a multidimensional construct that includes how one perceives, thinks, feels, and acts towards one’s body [12], all of which lies on a continuum from healthy body image perceptions (i.e., accurate and mostly positive) to unhealthy body image perceptions (i.e., inaccurate and mostly negative) [13,14]. Negative perception towards oneself due to having a considerably strong desire for thinness is a significant public health concern, particularly for young females. A study involving subjects from 26 countries revealed that body size dissatisfaction and having a strong desire for thinness were common attributes among females from high socio-economic status (SES) and among those who have been exposed to Western media [15]. Furthermore, a Malaysian study revealed that body size dissatisfaction contributes to higher body mass index (BMI)-for-age among young adolescents in Malaysia [16], whilst another local study found that negative body image was associated with disordered eating among adolescents [17]. The negative body image perception may have been established in childhood and adolescence, and may have worsened in their young adulthood through experiencing a challenging university life.

Disordered eating (DE) is defined as a constellation of unhealthy eating and weight-related behaviours and attitudes that do not meet the criteria for eating disorders, but that may have medical and/or psychological consequences [18]. The prevalence of DE was found to be rising in lower- and upper-middle-income countries in South-East Asia compared to other regions in the world [19]. For instance, 11.5% of students across South-East Asian countries were classified as being at risk for an eating disorder, which ranged from below 10% in Indonesia, Thailand, and Vietnam to 13.8% in Malaysia and 20.6% in Myanmar [19]. An excessive desire for thinness has been associated with DE including dieting, skipping meals, and unbalanced eating patterns. Consequently, DE has been associated with both short- and long-term health risks, including fatigue and hormonal abnormalities that may result in menstrual irregularities and decreased bone mineral density [20,21,22,23].

Studies have found DE to be confined to certain sections of SES, either high or low SES [19,24]. Furthermore, it was reported that relationships of DE with body image and socio-demographic characteristics vary by sex [25,26]. In female students, the most significant correlate of DE was found to be body shape concerns, whilst in male students, anxiety and concerns related to muscle size and shape were the most significant correlates of DE [25]. Although a prospective cohort study showed that BMI was correlated with DE in both sexes, the strength of correlation was higher in female students compared to males [26]. A previous study showed that there was an association between socio-demographic characteristics and DE in Malaysia [27] but the differences in the associations by gender have not been investigated in detail. Thus, the aim of the present study was to compare factors associated with DE in male and female university students in Malaysia. It is hypothesised that socio-demographic characteristics, BMI, body appreciation, body size satisfaction, body parts satisfaction, and depressive symptoms will make significant contributions towards DE and that the factors that associated with DE will be different between male and female university students in Malaysia.

## 2. Materials and Methods

### 2.1. Study Design and Participants

This is a cross-sectional study carried out in three public universities and four private universities in Kuala Lumpur and Selangor, Malaysia. A total of 716 students studying in one of the seven universities consented to take part in this study. Students enrolled in postgraduate studies, part-time students, and those who were pregnant were excluded from the present study. The height and weight of all participants were measured and they were asked to complete a paper questionnaire that contained questions on socio-demographic characteristics, eating attitudes, body image, and depressive symptoms.

### 2.2. Questionnaire on Socio-Demographic Characteristics

Socio-demographic characteristics that were assessed in this study included sex, age, ethnicity, and year of study. The university students who participated in the current study were from the three major ethnic groups in Malaysia, namely Malays, Chinese, and Indians. Other ethnic groups such as Indigenous people, Sabah Aborigines and Sarawak Aborigines are the minorities in Malaysia. The other ethnic group was chosen as the reference group in the current study which allows for comparison with other studies in Malaysia.

### 2.3. Eating Attitudes Test-26 (EAT-26)

The Eating Attitudes Test-26 (EAT-26) [28] is a 26-item test that can be used to assess DE habits of respondents. The test consists of three sub-items, which are dieting, bulimia and food preoccupation, and oral control. The items are rated using a 6-point Likert scale: “(1) for always”; “(2) for usually”; “(3) for often”; “(4) for sometimes”; “(5) for rarely”, and “(6) for never”. For each item in the test (except item 26), the responses “sometimes”, “rarely”, and “never” receive a score of 0 whilst the responses “always”, “usually”, and “often” receive a score of 3, 2, and 1, respectively. In contrast, item 26 is scored in a reversed manner. Scale scores are the sum of all items in each sub-item and the possible total EAT-26 score, which can range from 0 to 78. Respondents who scored 20 or more, were deemed to a high level of concern about dieting, body weight, or problematic eating behaviours, which were associated with eating disorders. Therefore, these individuals were considered to have DE or to be at risk (AR) of developing eating disorders [28]. Meanwhile, respondents who scored less than 20 were considered as not having any symptoms of eating disorder or as not at risk (NAR) [28]. The Cronbach’s alpha for the EAT-26 in the current study was 0.779 (0.763 in males and 0.782 in females).

### 2.4. Body Appreciation Scale (BAS)

The Body Appreciation Scale (BAS) [29] is a 13-item test used to assess an individual’s favourable opinions of his/her body regardless of actual appearance and accepting his/her body despite their perceived shortcomings of their own appearance. The items are rated on a 5-point Likert scale (1 = never, 2 = seldom, 3 = sometimes, 4 = often, 5 = always). The possible total score ranges from 13 to 65. A higher score indicates greater body appreciation. The validated Malay version of the BAS [30] was also used in this study. The Cronbach’s alpha for the BAS in the present study was 0.915 (0.906 in males and 0.919 in females).

### 2.5. Contour Drawing Rating Scale (CDRS)

The Contour Drawing Rating Scale (CDRS) [31] was used to assess body size perception of the participants. It comprises nine ordered male and nine ordered female figures. Figures 1–3 represent underweight, Figures 4–6 represent normal weight, and Figures 7–9 represent overweight and obese weight. The respondents were required to select a figure that they felt best represented their current body size as well as choose a figure that they most desired to have. Each figure is given a score ranging from 1 to 9. The body size discrepancy score, which represents the degree of body size dissatisfaction, was calculated by deducting the score of the ideal body size from the score of current body size. A higher body size discrepancy score indicates a higher level of body size dissatisfaction.

### 2.6. Body Satisfaction Scale (BSS)

Body parts satisfaction was measured with a 16-item version of the Body Satisfaction Scale (BSS) [32]. The respondents rated their satisfaction towards their own body parts (e.g., ankles, hips, thighs) on a 7-point scale, ranging from “very satisfied” to “very unsatisfied”. The possible total score of BSS ranges from 16 to 112. The higher the score, the higher the dissatisfaction of the respondents towards their body parts. The Cronbach’s alpha of the BSS scale was 0.961 for males, 0.937 for females, and 0.943 for the overall sample.

### 2.7. Center for Epidemiological Studies-Depressed Mood Scale (CES-D)

The 20-item Centre for Epidemiological Studies-Depressed Mood Scale (CES-D) [33] was used to measure symptoms, such as depressed moods, feelings of guilt and worthlessness, feelings of helplessness and hopelessness, psychomotor retardation, loss of appetite, and sleep disturbance. The responses within the scale were based on the frequency of occurrence of each symptom. The responses that could be chosen were “rarely or none of the time” (less than 1 day), “some or a little of the time” (1 to 2 days), “occasionally of a moderate amount of time” (3 to 4 days), and “most or all of the time” (5 to 7 days). The score for every response on a negative item ranged between 0 and 3; the scoring was reversed for positive items (items 4, 8, 12, and 16). The total CES-D score ranged between 0 and 60. A higher CES-D score indicated a higher depressive symptom, which increased the risk of depression. A sign of significant depressive symptoms was a score of 16 or more [33]. Cronbach’s alpha for the CES-D in this sample was 0.761 in overall, 0.749 in males and 0.765 in females.

### 2.8. Anthropometric Measurements

Body weight was measured to the nearest 0.1 kg with a digital weighing scale (SECA model 803, Hamburg, Germany). Height was measured using a portable stadiometer (SECA model 213, Hamburg, Germany) to the nearest 0.1 cm. Body mass index (BMI) was calculated as kg/m^2^ and categorised into four categories based on the WHO reference, namely underweight, normal weight, overweight, and obesity [34].

### 2.9. Statistical Analysis

Data were analysed using the IBM SPSS Statistics version 21 software (IBM SPSS Statistics, Inc., Chicago, IL, USA). Data were presented as mean and standard deviation (SD) for continuous variables and as frequencies and percentages for categorical variables. Independent student *t*-test was used to determine the mean differences of continuous variables, whilst the Chi-square test was used to determine the association of categorical variables between male and female university students. Pearson and Spearman correlation analyses between gender and DE were also performed. Hierarchical multiple regression analyses were also conducted to examine whether psychological factors contributed significantly to the prediction of individual DE problem, after accounting for the predictive contribution of previously identified BMI and socio-demographic variables for males and females, respectively. Each regression consisted of three separate blocks of variables, with the independent variables in each block entered sequentially. This approach reduced the effects of multicollinearity as it measures the degree to which each set of independent variables account for variances of the dependent variable, without the influence of subsequent blocks of variables. In each regression model, age, ethnicity, and type of university representing the socio-demographic variables were entered in the first block. BMI was entered in the second block. Next, the predictive contribution of psychological factors, such as body appreciation, body size satisfaction, body parts satisfaction, and depression, were entered as these factors had previously been associated with a DE problem. The R-square changed for each block is tested with an *F*-test. The significance level was set at <0.05 for all analyses.

### 2.10. Ethical Approval and Consent

The study was approved by the Universiti Teknologi MARA (UiTM) Research Ethics Committee (Ref: 600-RMI (5/1/6)). The researchers explained about the study protocol to potential respondents and written informed consent was obtained from all respondents prior to participating in the study.

## 3. Results

### 3.1. Characteristics of University Students

Table 1 shows the socio-demographic characteristics of the respondents. A total of 716 university students consented to participate in this study. The mean age of the respondents was 21.59 ± 1.42 (Table 1). The majority of the respondents were females (72.6%). In terms of ethnicity, more than half were Chinese (55.0%) and this was followed by Malay (34.9%), Indian (7.4%), and other ethnicities (2.7%). Most of the participants (45.8%) were enrolled in the second year of a Bachelor’s degree at their respective universities.

As shown in Table 2, there was a significant difference in BMI between the male and female respondents, where the males had a higher BMI compared to the females (t = 4.38, *p* < 0.05). The results show that there were more male students (21.9%) who were overweight and obese when compared to female students (13.3%) (χ^2^ = 14.37, *p* < 0.05). In addition, there were more female students (16.7%) who were underweight when compared to male students (8.2%) (Table 2). The female respondents obtained a higher mean score in EAT-26 test as compared to males (t = −3.23, *p* < 0.05) and there was a significantly (χ^2^ = 8.16, *p* < 0.05) higher percentage of female students (22.9%) who were engaged in DE when compared to males (13.3%) (Table 2). The results from the CDRS showed that the female participants had a lower mean body size discrepancy score compared to the male students (t = 10.77, *p* < 0.05). There were no significant differences in terms of mean scores of body appreciation and body parts satisfaction between males and females. There was also no significant sex difference in depressive symptom scores (Table 2).

### 3.2. Correlates of BMI, Socio-Demographic, and Psychological Factors with Disordered Eating

As shown in Table 3, DE was found to be significantly associated with ethnicity (r_s_ = −0.104, *p* < 0.05), BMI (r = 0.094, *p* < 0.05), body parts satisfaction (r = 0.079, *p* < 0.05), depressive symptoms (r = 0.259, *p* < 0.01), body appreciation (r = −0.189, *p* < 0.01), and body size satisfaction (r = −0.164, *p* < 0.01). When stratified by gender, DE was positively correlated with depressive symptoms, but inversely correlated with body appreciation, for both male and female participants. Among female participants, DE was also negatively correlated with ethnicity (r_s_ = −0.121, *p* < 0.05) and body size satisfaction (r = −0.143, *p* < 0.01), but positively correlated with BMI (r = 0.109, *p* < 0.05).

### 3.3. Multivariate Prediction of Disordered Eating

Table 4 shows the results of hierarchical regression analysis for DE among male university students. In Block 1, the results from male Indian university students were compared to other ethnic groups (β = 0.223, *p* < 0.01). The inclusion of BMI in Block 2 did not contribute significantly to the prediction of DE. When psychological factors, including body parts satisfaction, depressive symptoms, body appreciation, and body size satisfaction, were entered in Block 3, these appear to contribute significantly to the individual prediction of DE, accounting for a further 9.4% increase in the prediction of DE (ΔR^2^ = 0.094, *p* < 0.01). In the final model, it was shown that depressive symptoms contributed to the largest number of variances explained (β = 0.228, *p* < 0.01). Taken together, these variables explained 16.4% of the variances in DE model of male university students.

Table 5 shows the results of hierarchical regression analysis for DE among female university students. The results of public university were compared to private university in Block 1 (β = 0.092, *p* < 0.05) and Block 2 (β = 0.088, *p* < 0.05) and appear to give significant contributions to the variation in DE of female university students (Table 5). In Block 3, psychological factors, including body parts satisfaction, depressive symptoms, body appreciation, and body size satisfaction, contributed significantly to the DE model, accounting for a further 8.4% increase in the prediction of DE (ΔR^2^ = 0.084, *p* < 0.001). In the final model, depressive symptoms contributed the largest amount of explained variance (β = 0.214, *p* < 0.001), followed by body size satisfaction (β = −0.145, *p* < 0.01) and body appreciation (β = −0.101, *p* < 0.05). Taken together, these variables explained 10.2% of the variances in DE among female university students.

Overall, the results from Indian university students were compared to results from other ethnic groups (Block 1: β = 0.139, *p* < 0.05; Block 2: β = 0.146, *p* < 0.05) and the results from public university were compared to private university (Block 1: β = 0.094, *p* < 0.05; Block 2: β = 0.089, *p* < 0.05) as these appeared to be significant predictors of DE in the first two blocks (Table 6). Psychological factors including body parts satisfaction, depressive symptoms, body appreciation, and body size satisfaction that were entered in Block 3, contributed significantly to the prediction of DE, with a further 9.7% increase in the prediction of individual DE (ΔR^2^ = 0.097, *p* < 0.001). In the final model, depressive symptoms contributed the largest amount of explained variances (β = 0.226, *p* < 0.001), followed by lower body size satisfaction (β = −0.153, *p* < 0.001), poorer body appreciation (β = –0.114, *p* < 0.01), and Malay university students as compared to other ethnic groups (β = 0.210, *p* < 0.05). Taken together, these variables accounted for 15.8% of explained variances in DE among overall university students.

## 4. Discussion

In the present study, a higher proportion of female university students (22.9%) were found to have DE when compared to male students (13.3%). The findings were similar to the results of the Wave III of the National Longitudinal Study of Adolescent to Adult Health (Add Health), which also found a higher proportion of female young adults to be engaged in unhealthy weight control behaviours, binge eating behaviour, and being diagnosed with eating disorders when compared to their male counterparts [35]. The DE assessments that are related to concerns on being overweight, dieting, vomiting, and skipping meals, served as a proxy for risk of eating disorders based on thin-ideal body size-oriented goals, which may be more relevant to females. This is because females are reported to usually desire a thinner shape or smaller body size compared to males, who desire a leaner and more muscular shape or larger body size [36,37]. A recent study among university students from the United Arab Emirates (UAE) also found that a higher proportion of females were dissatisfied with their body size, whereby females desired to lose weight and preferred diet to exercise, and males desired to gain weight and preferred exercise to diet [38]. Future DE assessment may need to consider examining compulsive exercise [39] and androgenic anabolic steroid use [40], which may be more prevalent among male young adults. In the present study, higher body size dissatisfaction and poorer body appreciation contributed significantly to DE in female students, but not in male students. A previous study conducted in Malaysia had found that the higher acceptances towards body image in female undergraduate Malaysian medical students were associated with higher susceptibility to eating disorders [41]. Body size dissatisfaction also predicted a higher risk of DE in urban females [42] and appears to contribute to binge eating behaviours among Malaysian adolescents [17]. In a sample of South-East Asian university students, it was found that students who perceived themselves as overweight had higher odds of DE when compared to students who had perceived themselves as having normal body weight [19]. Based on a review of 78 studies, Haynes and colleagues reported that those who perceived themselves as being overweight reported higher levels of DE than those who perceived themselves as normal weight [43]. The 15-year longitudinal project “Eating and Activity in Teens and Young Adults (EAT)” also revealed that body dissatisfaction predicted a wide range of DE outcomes, including dieting, unhealthy weight control behaviours, as well as binge eating, particularly in female participants [44]. Whilst many may practice DE to achieve their unrealistic slim ideal body size, DE may lead to more weight gain [43] and poorer overall health [45].

Malaysia is a multi-ethnic country with Malay, Chinese and Indian as the major ethnic groups. However, the current study found that ethnicity was not a significant predictor of DE among male or female students from different universities after adjustment for confounders. In other words, all ethnic groups were at risk of DE in university students. Contrary to the several local studies, ethnicity was significantly associated with disordered eating in Malaysian children and adolescents [16,17,27]. It might imply that university students are more concerned about their body image compared to their younger counterparts that resulted in DE across the ethnic groups. Thus, prospective cohort studies are needed to examine the changes of DE in different ethnic groups from childhood to early adulthood. In the present study, depressive symptoms were the strongest predictor of DE in both male and female university students. The association between depression and DE was demonstrated in the previous study, which showed that students with depressive symptoms were more likely to report DE when compared to students who did not have depressive symptoms (OR = 1.86, *p* < 0.001) [19]. In a sample of Malaysian female medical university students, although there was a positive relationship between depression and DE, a negative relationship was found between acceptance of body image and depression [41].

In a review by McBride and Cole [46], longitudinal relations between depressive symptoms and binge eating have been reported. Furthermore, a meta-analysis involving 30 longitudinal studies concluded that there was a bi-directional relationship between eating pathology (e.g., an eating disorder or disordered eating symptoms) and depression (e.g., a depressive disorder diagnosis or depressive symptoms) [47]. The outcomes conjointly give initial support for the view that DE increases the risk of depression, potentially as a result of failure to manage eating behaviours, for example dietary restraint and binge eating. This could in turn lead to failure to attain an idealised physique, which then may cause depression [48,49]. Over-valuation of body image predicted both DE and depressive symptoms [44]. Local studies found inconsistent findings in the mediational effects of depressive symptoms on the relationship between negative body image and DE [41,50]. A longitudinal study is needed to confirm the influence of body image and depressive symptoms on DE.

A key strength of the current study is to highlight both male and female Malaysian university students were facing DE problems, and different factors were associated with DE between males and females. The present study has several limitations to be taken into consideration. Firstly, this study is a cross-sectional survey and no causal relationship could be made. Secondly, this study involved university students in urban areas of Kuala Lumpur and Selangor. Therefore, the outcomes of the present study cannot be used to generalise all university students in Malaysia. Furthermore, self-reported questionnaires were used to obtain information from the participants, which could have led to biases and elicit more socially desirable responses from participants. Nonetheless, the questionnaires used are valid and reliable instruments and widely used by researchers, such as EAT-26, which is one of the most common measures for disordered eating or eating disorders risk.

In conclusion, the present study found that depressive symptoms were the only significant predictor for disordered eating among male students. Depressive symptoms were also the most significant predictor for disordered eating in female students, followed by body image perception and body appreciation. The present study adds to the body of knowledge in that there are gender differences in the factors associated with DE among Malaysian university students. The outcome of this study could be used as a basis to develop an intervention in preventing DE among university students. Promoting psychological well-being should be included as one of the key components in the prevention programme of DE among university students. For future research, it is suggested to determine the contributing factors of the different forms of DE, such as binge eating/binge eating disorder, loss of control eating, emotional eating, compulsive exercise, and androgenic anabolic steroid use. This is because the contributing factors might be different for different forms of DE and at the same time, they might also share similar factors.

## Figures and Tables

**Table 1:** Socio-demographic characteristics of the participants (*n* = 716).

Variables	*n* (%)/Mean ± SD		
	Male (*n* = 196)	Female (*n* = 520)	Total (*n* = 716)
Age (years)	21.64 ± 1.47	21.57 ± 1.41	21.59 ± 0.45
Ethnicity			
Malay	90 (45.9)	160 (30.8)	250 (34.9)
Chinese	93 (47.4)	301 (57.9)	394 (55.0)
Indian	4 (2.0)	49 (9.4)	53 (7.4)
Others	9 (4.6)	10 (1.9)	19 (2.7)
Type of University			
Public	122 (62.2)	266 (51.2)	388 (54.2)
Private	74 (37.8)	254 (48.8)	328 (45.8)
Year of Study			
1	49 (25.3)	133 (25.6)	182 (25.5)
2	88 (45.4)	239 (46.0)	327 (45.8)
3	35 (18.0)	64 (12.3)	99 (13.9)
4	21 (10.8)	82 (15.8)	103 (14.4)
5	1 (0.5)	2 (0.4)	3 (0.4)

**Table 2:** Comparison of body mass index (BMI), disordered eating, body image perception, depressive symptoms, body appreciation, body image satisfaction, and self-esteem of university students according to sex (*n* = 716).

Variables	Mean ± SD			*t*	*n* (%)			χ^2^
	Male	Female	Total		Male	Female	Total	
BMI (kg/m^2^)	22.8 ± 3.9	21.4 ± 3.6	21.8 ± 3.7	4.38 *				14.37 *
• Underweight					16 (8.2)	87 (16.7)	103 (14.4)	
• Normal					137 (69.9)	364 (70.0)	501 (70.0)	
• Overweight					30 (15.3)	51 (9.8)	81 (11.3)	
• Obesity					13 (6.6)	18 (3.5)	31 (4.3)	
Disordered Eating, EAT-26	11.25 ± 7.97	13.49 ± 9.13	12.88 ± 8.88	−3.23 *				8.16 *
• At risk (AR)					26 (13.3)	119 (22.9)	145 (20.3)	
• Not at risk (NAR)					170 (86.7)	401 (77.1)	571 (79.7)	
Body Size Satisfaction, CDRS	−0.05 ± 1.06	−1.08 ± 1.32	−0.79 ± 1.33	10.77 *				
Depressive Symptoms, CES-D	17.56 ± 7.57	17.81 ± 8.04	17.74 ± 7.91	−0.38				2.16
• No depression					25 (12.8)	69 (13.3)	94 (13.1)	
• Mild depression					73 (37.2)	164 (31.5)	237 (33.1)	
• Moderate depression					67 (34.2)	198 (38.1)	265 (37.0)	
• Severe depression					31 (15.8)	89 (17.1)	120 (16.8)	
Body appreciation, BAS	3.80 ± 0.67	3.80 ± 0.71	3.80 ± 0.70	−0.01				
Body parts satisfaction, BSS	41.68 ± 1.31	43.14 ± 0.79	42.74 ± 18.10	−1.00				

BAS: Body Appreciation Scale; BSS: Body Satisfaction Scale; BMI: body mass index; CDRS: Contour Drawing Rating Scale: EAT-26: Eating Attitudes Test-26; CES-D: Center for Epidemiological Studies-Depressed Mood Scale. Classification criteria of BMI: Underweight: <18.5; Normal: 18.5–24.9; Overweight: 25.0–29.9; Obesity: ≥30. Note: * *p* < 0.05.

**Table 3:** Correlates of disordered eating with body mass index (BMI), socio-demographic characteristics, and psychological factors of university students.

Variables	Disordered Eating (EAT-26)		
	Males (*n* = 196)	Females (*n* = 520)	All (*n* = 716)
Age ^a^	−0.047	−0.048	−0.050
Ethnicity ^b^	−0.106	−0.121 *	−0.104 *
Type of university ^b^	−0.028	0.050	0.042
BMI ^a^	0.131	0.109 *	0.094 *
Body parts satisfaction ^a^	0.089	0.072	0.079 *
Depressive symptoms ^a^	0.289 **	0.250 **	0.259 **
Body appreciation ^a^	−0.233 **	−0.178 **	−0.189 **
Body size satisfaction ^a^	−0.103	−0.143 **	−0.164 **

Note: * *p* < 0.05, ** *p* < 0.01. **^a^** Pearson correlation coefficient (r); **^b^** Spearman correlation coefficient (r_s_).

**Table 4:** Hierarchical multiple regression analyses for individual prediction of disordered eating (EAT-26) from body mass index, socio-demographic characteristics, and personal variables in males (*n* = 196).

Variables Predicting Individual DE	B	SE	β	t	R	R^2^	Δ R^2^
Block 1					0.245	0.060	0.060 *
Age	−0.291	0.393	−0.054	−0.741			
Ethnicity:							
Malay	4.801	2.763	0.301	1.738			
Chinese	2.099	2.746	0.132	0.765			
Indian	12.521	4.733	0.223	2.645 **			
Others (Ref.)							
Type of university:							
Public	0.833	1.291	0.051	0.645			
Private (Ref.)							
Block 2					0.265	0.070	0.010
Age	−0.383	0.397	−0.071	−0.966			
Ethnicity:							
Malay	4.511	2.762	0.283	1.633			
Chinese	2.182	2.738	0.137	0.797			
Indian	11.988	4.734	0.213	2.532 *			
Others (Ref.)							
Type of university:							
Public	0.574	1.300	0.035	0.441			
Private (Ref.)							
BMI	0.217	0.150	0.106	1.451			
Block 3					0.405	0.164	0.094 **
Age	−0.243	0.385	−0.045	−0.631			
Ethnicity:							
Malay	4.018	2.672	0.252	1.504			
Chinese	1.193	2.691	0.075	0.443			
Indian	8.763	4.649	0.156	1.885			
Others (Ref.)							
Type of university:							
Public	0.503	1.280	0.031	0.393			
Private (Ref.)							
BMI	0.503	0.151	0.025	0.341			
Body parts satisfaction	0.014	0.035	0.033	0.412			
Depressive symptoms	0.240	0.080	0.228	3.012 **			
Body appreciation	−1.871	0.963	−0.158	−1.942			
Body size satisfaction	−0.677	0.528	−0.090	−1.283			

Note: * *p* < 0.05, ** *p* < 0.01.

**Table 5:** Hierarchical multiple regression analyses for individual prediction of disordered eating (EAT-26) from body mass index, socio-demographic, and personal variables in females (*n* = 520).

Variables Predicting Individual DE	B	SE	β	t	R	R^2^	Δ R^2^
Block 1					0.261	0.068	0.068 ***
Age	−0.406	0.279	−0.063	−1.458			
Ethnicity:							
Malay	1.140	2.905	0.058	0.392			
Chinese	−3.638	2.860	−0.197	−1.272			
Indian	1.000	3.092	0.032	0.323			
Others (Ref.)							
Type of university:							
Public	1.686	0.792	0.092	2.129 *			
Private (Ref.)							
Block 2					0.267	0.071	0.003
Age	−0.429	0.279	−0.066	−1.537			
Ethnicity:							
Malay	1.404	2.911	0.071	0.482			
Chinese	−3.108	2.890	−0.168	−1.075			
Indian	1.386	3.106	0.044	0.446			
Others (Ref.)							
Type of university:							
Public	1.608	0.794	0.088	2.025 *			
Private (Ref.)							
BMI	0.139	0.112	0.055	1.242			
Block 3					0.394	0.155	0.084 ***
Age	−0.442	0.270	−0.068	−1.638			
Ethnicity:							
Malay	2.912	2.809	0.147	1.037			
Chinese	−2.533	2.820	−0.137	−0.898			
Indian	2.220	3.001	0.071	0.740			
Others (Ref.)							
Type of university:							
Public	1.223	0.780	0.067	1.568			
Private (Ref.)							
BMI	−0.210	0.137	−0.083	−1.527			
Body parts satisfaction	−0.031	0.025	−0.062	1.238			
Depressive symptoms	0.244	0.054	0.214	4.522 ***			
Body appreciation	−1.295	0.635	−0.101	−2.039 *			
Body size satisfaction	−1.000	0.384	−0.145	−2.605 **			

Note: * *p* < 0.05, ** *p* < 0.01, *** *p* < 0.001.

**Table 6:** Hierarchical multiple regression analyses for individual prediction of disordered eating (EAT-26) from body mass index, socio-demographic, and personal variables in overall model (*n* = 716).

Variables Predicting Individual DE	B	SE	β	t	R	R^2^	Δ R^2^
Block 1					0.241	0.058	0.058 ***
Age	−0.431	0.229	−0.069	−1.880			
Ethnicity:							
Malay	3.384	2.065	0.182	1.639			
Chinese	−0.616	2.034	−0.035	−0.303			
Indian	4.722	2.316	0.139	2.039 *			
Others (Ref.)							
Type of university:							
Public	1.677	0.675	0.094	2.485 *			
Private (Ref.)							
Block 2					0.247	0.061	0.003
Age	−0.459	0.230	−0.074	−1.999 *			
Ethnicity:							
Malay	3.462	2.064	0.186	1.677			
Chinese	−0.280	2.045	−0.016	−0.137			
Indian	4.936	2.319	0.146	2.129 *			
Others (Ref.)							
Type of university:							
Public	1.592	0.677	0.089	2.353 *			
Private (Ref.)							
BMI	0.131	0.090	0.055	1.462			
Block 3					0.398	0.158	0.097 ***
Age	−0.418	0.220	−0.067	−1.905			
Ethnicity:							
Malay	3.907	1.968	0.210	1.985 *			
Chinese	−0.650	1.975	−0.036	−0.329			
Indian	4.270	2.222	0.126	1.921			
Others (Ref.)							
Type of university:							
Public	1.223	0.656	0.069	1.866			
Private (Ref.)							
BMI	−0.144	0.096	−0.061	−1.505			
Body parts satisfaction	−0.022	0.020	−0.046	1.106			
Depressive symptoms	0.254	0.044	0.226	5.746 ***			
Body appreciation	−1.446	0.526	−0.114	−2.749 **			
Body size satisfaction	−1.016	0.266	−0.153	−3.816 ***			

Note: * *p* < 0.05, ** *p* < 0.01, *** *p* < 0.001.

## Abbreviations

AR	At-risk
BAS	Body Appreciation Scale
BMI	Body Mass index
BSS	Body Satisfaction Scale
CES-D	Centre for Epidemiological Studies-Depressed Mood Scale
CDRS	Contour Drawing Rating Scale
DE	Disordered eating
EAT-26	Eating Attitudes Test-26
NAR	Not-at-risk
SD	Standard deviation
SES	Socio-economic status
SPSS	Statistical Package for the Social Sciences
WHO	World Health Organization
